# Music activities that most elicit interest and pleasure among older people with mild-to-moderate dementia: a qualitative multiple-case study in Italy

**DOI:** 10.3389/fnagi.2026.1873875

**Published:** 2026-09-09

**Authors:** Sara Santini, Alessandra Merizzi, Cristina Vesprini, Gabriele Morettini, Sabrina Quattrini

**Affiliations:** 1 Center for Socio-Economic Research on Aging, IRCCS INRCA-National Institute of Health and Science on Aging, Ancona, Italy; 2 AST Fermo, Medical Doctor Primary Health Care – Social and Health District, Fermo, Italy

**Keywords:** cognitive impairment, dementia, ideal types, interest, multiple qualitative case-study, music activities, non-pharmacological intervention, pleasure

## Abstract

Literature has well documented the impact of music on the mood of older adults with dementia and underscored that the pleasantness of activities may increase the intervention’s efficacy. Nevertheless, it is unclear which music activities may be more effective in triggering interest and pleasure in older adults with mild-to-moderate dementia. Recently, SOUND, a novel and original music-based non-pharmacological intervention, was conducted in Italy with 13 older people aged over 70 years with mild-to-moderate dementia who attended a day-care center. Participants’ interest and pleasure were rated and ranked using the Apparent Affect Rating Scale. Field notes were analyzed to develop ideal types of older people with mild-to-moderate dementia who participated in the intervention. Results showed that listening/attention/coordination activities and percussion/rhythmic activities were associated with greater observed interest and pleasure among older people with dementia, compared with elicitive/narrative activities and singing activities. The pleasantness and interest elicited by activities might be influenced by the educational background and music training of facilitators, participants’ cognitive profiles, and the social dynamics of the sessions. Five ideal types of older people with moderate dementia were identified as a potential tool to help dementia care professionals and musicians group participants and choose music activities to maximize the benefit of the intervention. Further research is needed to understand how music activities, from being pleasant and interesting, may become a source of stress and discomfort and how ideal types could inform the design of future interventions.

## Background and objectives

Dementia is a progressive neurodegenerative condition characterized by a decline in cognitive functions such as memory, attention, executive functioning, and language, often accompanied by neuropsychiatric symptoms including depression, anxiety, apathy, and agitation (Li et al., 2022). These symptoms substantially affect the quality of life not only of people living with dementia but also of their caregivers and healthcare professionals. In addition to cognitive deterioration, individuals frequently experience emotional dysregulation, with overlapping neuropsychiatric and mood disturbances, which further complicate care and management (Skoda et al., 2026).

In this context, non-pharmacological interventions have increasingly gained attention as complementary approaches to conventional medical treatments. Among these, music-based interventions have been widely investigated because of their potential to influence emotional, cognitive, and social domains.

Music appears to be very promising in reducing and countering neuropsychiatric symptoms (Lin et al., 2023), depression and anxiety (Gómez-Gallego and Gómez-García, 2017; Ray and Götell, 2018; Zhang et al., 2023), improving cognitive (Brancatisano et al., 2019; Ito et al., 2022; Jiménez-Palomares et al., 2024; Lin et al., 2023) and executive functions, and promoting wellbeing and quality of life in older people living with dementia (OPD) (Ray and Götell, 2018; Santini et al., 2025).

Music interventions can be broadly classified into active and receptive approaches. Active music interventions involve direct participation in the production of sound, such as singing or playing instruments, while receptive interventions are based on listening and guided experiences, including relaxation and reminiscence. As detailed below, evidence suggests that these two approaches may engage individuals differently.

Gómez-Gallego et al. (2021) indeed pointed out that active music interventions (AMIs) may improve cognition, behavior, and the functional state of older adults with dementia to a greater extent than receptive music interventions (RMI) and usual care provided in care facilities, while RMI may have a stabilizing effect on behavioral symptoms such as wandering and anger. Similarly, Dorris et al. (2021) argue that active music-making activities are the “key ingredient” for eliciting cognitive functions in people with mild-to-moderate dementia.

The RMI may function as a relaxation strategy, potentially contributing to sustained long-term benefits, whereas AMI can promote participants’ engagement through social interaction and may generate more immediate therapeutic effects (Matziorinis and Koelsch, 2022). Music, when used as a means of reminiscence, may enable OPD to recall moments dear to them and strengthen their personal identity (Matziorinis and Koelsch, 2022).

Group AMI, including choral singing and drumming, may reduce mental distress, depression, and anxiety in OPD, while improving individual and social wellbeing (Fancourt and Finn, 2019). Furthermore, the use of percussion instruments can reduce anxiety (Liu et al., 2021), while rhythmic auditory stimulation-based movement training can improve general cognition, memory, attention, and executive functions in patients with cognitive impairment (Wang et al., 2024). On the other hand, a literature review indicates that music-based techniques enhance behavioral and cognitive outcomes regardless of the type of approach (Leggieri et al., 2019).

Additionally, McMahon et al. (2023) state that shared music activities, whether active or receptive, are effective in fostering connections between older adults with dementia and their caregivers.

Recent meta-analytic data and large-scale randomized controlled trials (RCTs) indicate a clear divergence in outcomes based on the size of music interventions with people living with dementia. As part of the international MIDDEL mega-trial, Bloska (2025) conducted a video-analytic study differentiating structured group music therapy (GMT), characterized by active improvisation and collective instrument playing, from large-group community singing in terms of their respective interactional dynamics. These group dynamics are hypothesized to stimulate collective emotional convergence and social bonding, which might support resilient psychosocial functioning in institutionalized cohorts (Hatfield et al., 1993). Group-based formats are particularly powerful in mitigating externalizing neuropsychiatric symptoms. Moreover, collective AMIs, such as group singing and rhythmic percussion, seem to yield superior outcomes in reducing behavioral disturbances, including verbal aggression, wandering, and social isolation (Mulia, 2025). The same study also noted that individual interventions consistently yield greater improvements in direct cognitive performance metrics and targeted mood stabilization, as they minimize the sensory overload or confusion that can sometimes occur in group settings for patients with advanced neurodegeneration (Mulia, 2025).

Conversely, individual music interventions demonstrate distinct clinical utility, particularly for transient cognitive stabilization and acute anxiety management. Systematic reviews indicate that individual, person-centered playlists, tailored to a patient’s biographical and musical history, show marked superiority in reducing acute, situational agitation and anxiety during high-stress care routines, such as bathing or clinical examinations (Berkovic et al., 2024). The literature, therefore, seems to emphasize that group and individual music interventions should not be viewed as mutually exclusive but as complementary components of a comprehensive, person-centered neurorehabilitation framework.

Neurobiologically, music engages the brain’s reward system (Salimpoor et al., 2011). Under incentive-sensitization theory (IST) (Berridge and Robinson, 2016; Robinson and Berridge, 1993), this reward is split into dopamine-driven “wanting” (motivation) and non-dopaminergic “liking” (hedonic pleasure). Additionally, musical reward relies on a “tripartite network” where the hypothalamus connects pleasure to autonomic and neuroendocrine responses (Bowling et al., 2019, 2022). This core node couples temporally with auditory, frontal, insular, amygdalar, and hippocampal regions (Salimpoor et al., 2013; Koelsch, 2020) to integrate sensory processing, emotion, and memory (Bowling, 2023).

Such evidence suggests that pleasurable and meaningful musical experiences not only provide hedonic pleasure but can also engage motivational circuits that enhance attention, arousal, and readiness to engage.

Moreover, recent empirical evidence indicates that regular exposure to individuals’ preferred music can modulate functional connectivity between auditory regions and reward networks, thereby offering a plausible neural mechanism for cognitive benefit in older adults. In a longitudinal 8-week receptive music-based intervention, older adults who listened to self-selected pleasurable music showed increased connectivity between auditory cortices and medial prefrontal regions, and greater striatal representation of musical stimuli after training (Quinci et al., 2022).

Considering the above, the level of pleasantness and interest elicited by music activities for OPD may influence their participation and, consequently, improvements in cognition and wellbeing, as well as the overall effectiveness of the intervention. Therefore, understanding which music activities can elicit participants’ pleasantness and interest may help to design more effective non-pharmacological music-based interventions.

This study, based on the outcomes of the SOUND music-based non-pharmacological intervention (Santini et al., 2025), is aimed to identify which music activities were more successful in stimulating interest and pleasure in OPD during the intervention, through a qualitative approach involving 13 Italian older adults with mild-to-moderate dementia.

## Research design and methods

### Study design

In 2023, the SOUND quasi-experimental mixed-methods study was designed (Santini et al., 2024) to test the impact of an original music-based intervention on the wellbeing, mood, cognition, and behavior of a sample of OPD living in Italy, Portugal, and Romania (Santini et al., 2025).

Within SOUND, this study aimed to answer the following research question: Which music activities stimulated pleasure and interest in the Italian participants of the SOUND intervention?

For this purpose, we adopted a multiple-case mixed-method design in which each participant was conceptualized as a “case” (Stake, 2005), and any verbal and non-verbal signs of interest and pleasure were captured as reactions to the activities proposed during the intervention. Case studies are particularly suited for understanding the internal dynamics of change processes, and multiple cases can help examine how contextual factors influence individuals’ reactions and interventions (Yin, 2009; Wensing et al., 2005).

### The intervention co-design

Literature suggests considering participants’ characteristics and music preferences to conceptualize music-based interventions for people with dementia (Reschke-Hernández et al., 2023), as well-known and personally meaningful music can be more effective in stimulating cognition and wellbeing in OPD by promoting greater emotional and cognitive engagement (Jakob et al., 2024). Thus, the SOUND intervention was preceded by a co-design phase, which included the collection of OPD biographical information and music preferences through family caregivers and, whenever possible, through patients (by showing pictures and playing songs to stimulate thinking and memory), to ensure personalization of the intervention and a person-centered approach to research. The activities were planned according to the objective of every session (e.g., verbal fluency, memory, and coordination) and were chosen based on participants’ actual mental and physical condition, personal music preferences, fears, and triggering factors (identified using a biographical sheet).

### Music activities

The Italian SOUND intervention encompassed 12 sessions, each including active and receptive music activities delivered two times a week over six consecutive weeks, between October and December 2023. The same intervention was carried out in two groups in parallel. The level of complexity of activities increased progressively throughout the program.

Each session lasted about 45 min and was divided into four phases: (1) welcoming; (2) opening activity; (3) 3–5 main activities depending on the length and intensity of each one; and (4) closing activity.

During every phase, except for phase 3, only one activity was proposed. Each session had a main objective with corresponding activities delivered in the same order in both groups so that they could be compared in data analysis, although a certain degree of customization was allowed to accommodate participants’ music preferences. For example, the tunes/songs did not have to be the same in the two groups, but the genre was consistent across groups.

Activities were developed based on (a) participants’ personal music preferences and life stories; (b) their physical health condition; and (c) the objective of the stimulation activities (e.g., verbal fluency, memory, coordination, auditory stimulation, attention stimulation, and coordination between body movements and listening to music). Activities were also tailored to accommodate OPD’s attitudes, fears, emotional sensitivities, and potential triggers, drawing on information from their personal history and current circumstances.

Once OPD’s music preferences had been identified, the selection of tunes included traditional themes, as this enhanced familiarity and resonance for participants.

Despite the minor adjustments described, the OPD in the two groups undertook the same activities to achieve the same objectives, following the same SOUND method, on which the two facilitators had been trained over 22 h. This ensured the methodological consistency of the intervention (Quattrini et al., 2024).

### Group composition: OPD, observers, and facilitators

Participants were split into groups only for organizational reasons, i.e., eight OPD attended the day-care center on Mondays and Wednesdays, and seven on Tuesdays and Thursdays. Moreover, the maximum number of participants per group was set at eight in order to ensure active involvement in the activities, facilitate support, and monitor reactions and behaviors by internal observers.

The latter were care professionals belonging to the day-care center, who sat next to the OPD, one for every two older adults, to support participants during the intervention in a non-intrusive way and notify the facilitator in case of observed discomfort. Researchers sat outside of the circle as external observers. They completed the AARS and took notes, as described in Santini et al. (2025).

The activities were led by two female facilitators, both trained in the method (Quattrini et al., 2024), one for each group. One facilitator was a professional educator working at the day-care center hosting the intervention, with a background as a musician (traverse flute player). The other facilitator was a singer, performer, and singing teacher with experience in delivering music activities for people with autism. The facilitators proposed the activities to OPD, arranged in a circle in a responsorial style, led the activities, welcomed and repeated any spontaneous and unexpected input from the participants, and then proposed it back to the whole circle.

### Sampling: inclusion criteria and recruitment strategies

The study recruited 15 OPD from each participating country. A purposive sampling strategy (Luborsky and Rubinstein, 1995) was used to identify individuals capable of providing rich, experience-based insights from an insider’s perspective. Italian participants were enrolled through the Alzheimer’s day-care center of the National Institute of Health and Science on Aging (INRCA), in Central Italy. The eligibility criteria required participants to be aged 65 years or older, to express interest in the study, and to have adequate vision, hearing, mobility (with or without assistive devices), and speech abilities (people with severe aphasia were excluded), to be able to understand simple instructions and to have a level of cognitive impairment ranging from mild to moderate. The severity of older adults’ cognitive impairment was assessed using the Montreal Cognitive Assessment (MoCA), which ranges from 0 (maximum cognitive impairment) to 30 (no cognitive impairment) (Nasreddine et al., 2005). Cognitive impairment was categorized as mild if the score was 18–25 (in this case, inclusion of the participant in the sample required a formal clinical diagnosis of mild dementia) and moderate if it was 10–17, according to the MoCA website (Nasreddine, 2026). Participants were screened before the start of the intervention by psychologists who administered the MoCA test face-to-face. Only individuals who provided written informed consent were enrolled in the intervention.

### Data collection tools and methods

To capture both objective behavioral durations and the contextual evolution of responses in older adults with dementia (OPD) during music sessions, a dual-observer approach was implemented. Every participant’s behavior was evaluated by an external observer and an internal observer, operating independently.

The external observer logged objective behavioral expressions of interest and pleasure strictly using the Observed Emotion Rating Scale (AARS) (Lawton et al., 1999).

The AARS aims to rate five emotions, i.e., pleasure, interest, fear/anxiety, anger, and sadness, and their duration on a 0–5 scale (0 = cannot tell; 1 = never; 2 = less than 16 s; 3 = 16–59 s; 4 = 1–2 min; 5 = more than 2 min) by observing the person for 5 min in three observation windows (from minute 5 to minute 10, then from minute 20 to minute 25, and from minute 35 to minute 40). For example, an OPD who appeared to experience pleasure for 16–59 s during the five observation minutes was rated 3 for pleasure, indicating that the activity was moderately pleasant for them. The three 5-min observation windows within each 45-min session were not fixed at rigid time intervals (e.g., 5–10, 20–25 min); instead, they were flexibly timed to align directly with the start and end of specific live music activities as they unfolded. When live observation covered only part of an activity’s duration, video recordings were retrospectively reviewed to evaluate and complete the AARS rating for the entire activity segment. In cases where more than one macro category occurred or overlapped within an observation window, independent AARS scores were assigned to each discrete activity segment based on its precise time boundaries, ensuring that ratings directly corresponded to individual macro categories. The internal observer recorded continuous narrative field notes capturing qualitative variations, subtle emotional nuances, and session-wide trajectories (e.g., observed increases or reductions in engagement over time).

The scores recorded through the AARS were complemented with notes taken by both internal observers (occasionally paired with additional field notes taken by external researchers), who captured participants’ attitudes and verbal and non-verbal expressions. The internal observers did not generate AARS ratings, but only produced field notes, where they transcribed what they observed as soon as the session ended, while external observers took written notes during the session. Inter-rater agreement statistics were therefore inapplicable to their contribution. The field notes for each participant were then compiled into a diary and subsequently verified by reviewing footage recorded by two cameras positioned at opposite corners of the circle during each session. Moreover, researchers consulted with dementia care professionals who, having a daily relationship with the participants, could help to correctly interpret certain behavioral manifestations and reduce the risk of misreading the observed non-verbal language. This helped to mitigate the risk of subjectivity and to capture incongruences between internal and external observers’ evaluations.

### Data analysis

#### Aggregation of SOUND activities

To avoid disproportionately weighting sessions that included a larger number of activities within the same category, a two-step aggregation procedure was adopted. First, for each participant, the ratings of all activities belonging to the same category within a given session were averaged, yielding a single session-level score for that activity category. Second, the session-level scores were averaged across all sessions in which that activity category was performed, resulting in one mean score per participant for each of the four activity categories. This procedure ensured that each session contributed equally to the participant’s overall score for a given activity category, regardless of the number of activities administered during that session.

Data were analyzed using descriptive statistics to summarize participant scores across the four music activity categories: listening/attention/coordination activities (LACA), percussion/rhythmic activities (PRA), elicitive/narrative activities (ENA), and singing activities (SA) (Table 1).

**Table 1:** Categorization of SOUND activities.

Macro category	Detailed type of activities
Singing activities (SA)	- Singing good morning with or without names - Singing bye-bye
Percussion/rhythmic activities (PRA)	- Percussion with sticks and musical instruments - Body percussion
Elicitive/narrative activities (ENA)	- Creating a story as a group - Listening to a background music track - Using images and/or markers
Listening/attention/coordination activities (LACA)	- Colored foulards - Colored cloth with or without plastic balls or leaves - Listening to calming music

It should be noted that the SA category was adopted for opening and closing sessions (and was sometimes shorter than the other activities, depending on the length of the proposed song). Moreover, the LACA category encompasses a degree of internal heterogeneity, combining multisensory and motor-coordination tasks (e.g., colored foulards, cloth with balls) with receptive auditory experiences (calming music). Because these sub-components engage distinct neurophysiological and phenomenological pathways, aggregate LACA scores reflect the combined effect of this activity cluster rather than any single constituent element.

#### Analysis of the AARS per SOUND macro category

The AARS scores, separately for the level of interest and pleasure recorded throughout the intervention, were considered. The average score obtained for each activity across the 12 sessions was calculated, excluding 0 values (missing data). An average score for each macro category of SOUND activities was calculated to obtain a classification from the highest (rank 1) to the lowest (rank 4) macro category in terms of effectiveness in stimulating either interest or pleasure.

Standard deviation (SD) and 95% confidence intervals (CIs) were computed for each activity’s mean interest and pleasure scores. The Wilcoxon signed-rank test was conducted for pairwise comparisons and to evaluate differences among the four categories of musical activity.

#### Field notes analysis

The field notes were transformed into narratives by extracting only the elements relevant to the study aims (Kutsche, 1998), then synthesized, and the resulting summaries were analyzed using qualitative content analysis (Forman and Damschroder, 2008; Hsieh and Shannon, 2005; Kohlbacher, 2006; Mayring, 2000). Units of analysis and coding units were defined from the smallest (single words) to the largest (whole diary). Each extracted fragment of text was coded following both a deductive and inductive approach and organized in a code system (Table 2). After reviewing the recorded sessions, the last author integrated the AARS scores assigned by the external observers with the qualitative field notes provided by the internal care professionals to develop an overall interpretation of each participant’s engagement and emotional responses. The first author independently reviewed these interpretations in light of the original observational material. Differences in interpretation were discussed until consensus was reached, with the second author involved when necessary to enhance the credibility and trustworthiness of the analysis.

**Table 2:** Code system.

Codes	Meaning	*N*
PLEASURE	Capturing any sign of pleasure, e.g., relaxed shoulders; open chest; leaning slightly toward the source of pleasure; reduced muscle tension; slow, fluid gestures	71
INTEREST/CONCENTRATION/CURIOSITY	Capturing participants’ attentive behavior through, e.g., sustained eye contact; eyebrows slightly raised; reduced blinking; head nodding; responsive facial expressions	87
HAPPINESS/AMUSEMENT/FUN	Picking up on any signs of fun and happiness, such as smiles, laughter, physical contact with other participants, and complicity dependent on achievements and external stimuli	78
PARTICIPATION/ENGAGEMENT	Capturing any sign of involvement and engagement in the proposed activities, e.g., responsiveness to the stimuli; playfulness; contributing ideas; opinions, or questions; building on others’ comments (“adding to what X said…”); using inclusive language (we, us, our); verbal affirmations: “yes,” “I agree,” “that makes sense”	34
ANXIETY/EMBARASSMENT	Grasping any sign of anxiety (e.g., restlessness: pacing, fidgeting, tapping hands or feet; shallow or fast breathing, frequent sighing; tense posture, clenched jaw or fists; sweating, blushing, or looking pale; avoiding eye contact or looking hyper-alert) and/or embarrassment (e.g., feeling uncomfortable or out of place)	28
PEACEFULNESS/JOY	Capturing verbal and non-verbal signs of quiet and peace of mind, e.g., serene and relaxed facial expression; regular breathing; seeking and giving gentle touches, as well as joy meant as an internal, long-lasting state of contentment, a mindset that makes people open to the proposed experiences, proactive and with a spirit of initiative	29
LOSS OF INTEREST/PLEASURE/INVOLVEMENT	Underlining any loss of interest/pleasure/involvement in the running activity as well as any temporary loss of attention	31
PROACTIVITY	Describing OPD who proposed a new exercise, a new movement, took the initiative and led the group, becoming the protagonist	21
SADNESS	Picking up on any signs (both verbal and non-verbal) of sadness elicited by the proposed activities (e.g., closed postures, head and jaw lowered toward the chest, curved trunk or shoulders, slowing of bodily movements, lowering the tone and intensity of the voice)	14
FRUSTRATION/BOREDOM	Capturing any verbal and non-verbal sign of frustration or annoyance (e.g., frowns, sighs and snorts, rolling of the eyes)	11
CRITICAL BEHAVIOR	Picking up on sarcastic comments; critical words and gazes; irony; general physical closed-off attitude; crossed arms	9
ANGER/IRRITATION	Verbal and non-verbal signs of aggressiveness with different intensities (e.g., screaming, raising the voice, motor restlessness; hyperreactivity to external stimuli; irritability; stereotyped and purposeless motor activity)	8
SELF-ESTEEM IMPROVEMENT	Highlighting any manifestation of an increase or improvement in participants’ self-esteem through words (e.g., “we are good!”), mimicry, and behavior expressing satisfaction with one’s performance	2
MASK-FACE	Grasping the lack of facial expression frequently observable in people living with dementia	2
Total		425

It is worth clarifying that, due to the natural variability in attendance among participants and observers across the 12 intervention sessions, different external observers assessed the same participant at different time points. Consequently, no duplicate ratings of the same observation were available, and statistics such as Cohen’s kappa or per cent agreement could not be calculated.

#### AARS and field notes triangulation

To capture both standardized performance and contextual depth, quantitative data from external observers using the AARS were integrated with qualitative field notes compiled by internal observers. The external AARS scores provided a structured benchmark for observable metrics, while the internal field notes offered critical context regarding participant dynamics and environmental nuances.

The analysis of the field notes, triangulated with that of the AARS, shaped the identification of activities that stimulated not only interest and pleasure in the participants but also happiness/amusement/fun, participation/engagement, peacefulness, proactivity, anxiety/embarrassment, and anger/irritation (Table 2).

Some quotations from the diaries are reported as a snapshot of the observed levels of interest, pleasure, and other emotions that coexisted or alternated during the observation time. The incongruences between AARS ratings and observations were discussed within the research team, and an agreed explanation was reached.

In the presentation of qualitative excerpts and summary tables (e.g., Table 3), these two data sources are juxtaposed to provide a comprehensive profile. Interpretive descriptors (such as “reduction” or “increase”) derive exclusively from the internal observers’ qualitative field notes, whereas temporal intervals (e.g., “16–59 s”) denote the objective AARS duration anchor recorded by the external observers and embedded in the observation report to facilitate data triangulation.

**Table 3:** Description of ideal types by case and excerpts.

Ideal types (OPD nos.)	Description	Excerpts
Body-driven (8 and 14)	They were especially stimulated by LACA and PRA, i.e., exercises that required moderate body involvement and coordination. They were collaborative and responsive to other types of activities, but their interest and attention suddenly waned with ENA	*He participates very actively in the hand/foot/music coordination exercise and is smiling* (OPD8) *During the story-telling activity with images, she shows a reduction in the level of interest* [Internal Observer note] *(AARS duration 16–59 s [External Observer]) and an increase in expressions of anxiety* (OPD14)
Just singing (10 and 13)	They were stimulated mostly by SA for interest and pleasure, while LACA was a source of stress and frustration as they frequently asked for reassurance on their performance. The singing was often used as an antidote to boredom: it represented a way to contain anxiety	*Today he is cheerful and enjoys almost all activities, while maintaining his preference for singing and listening to songs and is less interested in the spoken parts or the proposed movements* (OPD10)
Reflective (4 and 12)	They respond better to ENA than to any other activity. They enjoyed this kind of activity and were serene. On the contrary, PRA and SA did not seem to put them at ease, and they expressed signs of anxiety about the performance	*The story activity captured her interest and pleasure the most: she followed curiously, interacted with the facilitator, at times laughed and smiled and continued to seek dialog in a proactive way […] She becomes slightly anxious when the facilitator sings the words to her while waiting for her response* (OPD12)
Critics-anxious (1 and 11)	They rarely showed interest in any activity and did the exercises proposed with a critical attitude. They attempted to boycott activities through sarcasm and verbal criticism or aggressive attitudes, inciting the group to carry out activities different from those proposed by the facilitator. They sometimes showed anxiety, irritation, and sadness, especially during LACA	*In the exercise with the sticks, she begins saying: “She makes us feel more morons than we are”* (OPD1) *She tries to replace the facilitator by “leading” the movements of lifting the colored cloth up. She says: “everyone stands up: move your legs, arms, head, go! We want to have fun. She leaves the cloth when her proposal is not accepted by the facilitator”* (OPD11)
Enthusiastic (2, 3, 5, 6, 7)	They welcomed every type of activity with enthusiasm, interest, and enjoyment and did not show any signs of anxiety, embarrassment, or boredom. They felt comfortable and serene even in the face of difficulties or a drop in their attention. They often suggested variations to the exercise, and co-lead the activity, politely and without aggressiveness	*“He is involved, he seems attentive and concentrated, his gaze seems to indicate curiosity. During the repetition of a rhythm by clapping hands on the notes of “Il mio canto libero” by Lucio Battisti, he sings and seems to appreciate the song, which he immediately recognized.* *During the activity of the colored cloth, he shows a curious and attentive look.* *During the activity of the sheets, he reflects on what to write showing commitment and connection to reality and context.* *He plays the instrument in rhythm, appears so absorbed in the music that he sings with his eyes closed and moves his legs rhythmically”* (OPD3)

#### Ideal types identification

To capture the experiences of OPD in their complexity, participants’ Weberian “ideal types” (Weber, 1949) were drawn to create different “types” of older adults with dementia reacting to music prompts. In this study, the five ideal types represent an exploratory attempt to characterize OPD according to the musical activities and interactional features that appeared to elicit interest and pleasure during the intervention. Thus, the ideal types should be considered hypothesis-generating interpretative constructs rather than definitive categories, whose purpose is to provide a framework for reflecting on individual variability in responses to music-based activities and to inform future research investigating how personal and contextual factors may influence engagement in dementia care settings.

The results’ trustworthiness, i.e., credibility, dependability, and transferability, was achieved through internal process evaluations (Golafshani, 2003; Lincoln et al., 1985; Shenton, 2004). Credibility was achieved by adopting observation tools grounded in the existing literature on music and dementia (Lawton et al., 1999). Dependability was secured by establishing a meticulous and highly structured study design alongside rigorous operational planning, while confirmability was maintained through continuous self-reflection and investigator triangulation. The research team used a reflexive journal to systematically document personal assumptions, emerging thoughts, methodological decisions, and emotional responses; researchers also engaged in frequent collective discussions to neutralize potential preconceptions and safeguard the authenticity of the observed participants’ experiences.

### Ethical issues

The study was approved by the IRCCS INRCA Ethics Committee on 26 May 2023, with General Director Communication number 234 provided on 9 June 2023.

An informed consent form was given to all OPD and their family caregivers, and a signed copy was collected. The informed consent form described the study objectives, the list of activities, and the number of sessions and their length. It was also specified that the OPD could leave the intervention at any time without consequences and that the intervention had no risks or adverse effects on their health and wellbeing.

## Results

### Participants’ description

Thirteen OPD out of 15 were included in the analysis. OPD9 and OPD15 were considered drop-outs because, although they attended at least six sessions, they were not available to complete the questionnaire in the week of the data collection at the end of the intervention (T1): OPD9 could not be accompanied to the day-care center by the family carer for the entire week, and OPD15 had a stroke and did not attend the day-care center any more.

The sample had a mean age of 81.5 years and a medium-to-high educational level; six participants were male; eight had a partner/spouse, while the others were widowed; their professional background was quite varied (Table 4). According to the inclusion criteria, 11 of the 13 older adults had a MoCA score between 10 and 17, indicating a moderate cognitive impairment according to Nasreddine (2026), and two (OPD2 and OPD3) had scores of 19 and 18, respectively, but had received a formal clinical diagnosis of mild dementia from their treating clinicians. Therefore, their MoCA scores were not equated with MCI (18–25) as a nosological entity but with mild dementia, according to Nasreddine (2026).

**Table 4:** Sample description.

OPD	Age	Sex	Marital status	Education	Work before retirement	MoCA T0	Music*
1	83	F	Widowed	Primary school	Nurse	15	3
2	77	M	Married	Master’s degree	Bank director	19°	2
3	70	M	Married	Master’s degree	Director of the Regional Healthcare System	18°	1
4	84	M	Married	High school	Surveyor	10	1
5	79	M	Married	Primary school	Entrepreneur	15	3
6	81	F	Widowed	Secondary school	Trader	14	3
7	86	F	Married	High school	Administrative employee	12	2
8	83	M	Married	Master’s degree	Manager in a car company	14	1
9^§^	79	F	Married	High school	Bank clerk	11	1
10	81	M	Married	High school	Lift technician	10	1
11	84	F	Widowed	Primary school	Housewife	11	2
12	77	F	Married	Primary school	Trader	13	1
13	82	F	Widowed	High school	Housewife	13	4
14	93	F	Widowed	Secondary school	Nurse	10	1
15^§^	84	F	Widowed	Tertiary vocational diploma	Nursing Manager	10	1

*Historically, before the onset of the disease, how important was music in OPD’s life?

Possible answers: 1 = very important, 2 = moderately important, 3 = slightly important, 4 = not important.

^°^Cases who received a formal clinical diagnosis of mild dementia by their treating clinicians.

^§^Dropout.

The average MoCA score was 14.6 in the group comprising OPD1 to 8, and 11.4 in the group comprising OPD9 to 15 (drop-outs excluded), with a 3.2-point mean MoCA difference between the two subgroups.

Music was historically either very or moderately important in the lives of 9 of the 13 OPD (drop-outs excluded) (Table 4).

### Interest and pleasure by music activities: scoring and ranking

The analysis of the AARS scores by macro categories of activities (Table 5) shows that LACA and PRA elicited the greatest interest among OPD. Both had narrow CIs, reflecting strong and consistent levels of interest across participants.

**Table 5:** Interest mean score, standard deviation, standard error, and confidence interval.

Music activities	Mean	Ranking	SD	SE	95% CI	
Listening/attention/coordination activities (LACA)	4.2	1	0.48	0.13	3.93	4.50
Percussion/rhythmic activities (PRA)	4.2	2	0.61	0.17	3.80	4.54
Elicitive/narrative activities (ENA)	3.9	3	0.88	0.24	3.35	4.41
Singing activities (SA)	3.2	4	0.55	0.15	2.81	3.48

1 = the most interesting; 4 = the least interesting. Missing values were assigned when the participant was absent from a session.

Ranks 1, 2, and 3 do not significantly differ from each other (see Table 7, p > z).

Regarding the dimension of pleasure (Table 6), LACA (3.9) and PRA (3.8) elicited the highest levels of pleasure among OPD, followed by ENA (3.3) and SA (3.2).

**Table 6:** Pleasure mean score, standard deviation, standard error, and confidence interval.

Music activities	Mean	Ranking	SD	SE	95% CI	
Listening/attention/coordination activities (LACA)	3.9	1	0.65	0.18	3.52	4.31
Percussion/rhythmic activities (PRA)	3.8	2	0.86	0.24	3.30	4.34
Elicitive/narrative activities (ENA)	3.4	3	0.90	0.25	2.85	3.93
Singing activities (SA)	3.2	4	0.68	0.19	2.74	3.56

1 = the most interesting; 4 = the least interesting. Missing values were assigned when the participant was absent from a session.

Ranks 1 and 2 do not significantly differ from each other, nor do ranks 3 and 4 (see Table 7, *p* > *z*).

The relatively low standard error for both interest (0.13–0.24) and pleasure (0.18–0.25) indicates that OPD’s responses were fairly clustered around the mean, suggesting relatively consistent responses regarding LACA as the most and SA as the least interesting music activities.

The higher standard error in ENA for interest (0.24) and pleasure (0.25) and in PRA for pleasure (0.24) may indicate greater variability in the extent to which the activities elicited interest and pleasure in OPD compared with LACA and PRA, which achieved more homogeneous feedback from participants.

Pairwise comparisons using Wilcoxon signed-rank tests revealed specific differences across conditions rather than a strict uniform ranking (Table 7). For interest, SA scored significantly lower than LACA (*p* = 0.0019), PRA (*p* = 0.016), and ENA (*p* = 0.0192). However, no significant difference in interest was observed between LACA and PRA (*p* = 0.1618).

**Table 7:** Results of Wilcoxon signed-rank tests by interest and pleasure for music activities.

Music activities	Interest			Pleasure		
	*z*	*p* > *z*	*r* (effect size)	*z*	*p* > *z*	*r* (effect size)
SA vs. LACA	−3.112	0.0019*	0.61	−3.045	0.0023*	0.60
SA vs. PRA	−3.148	0.0016*	0.62	−3.15	0.0016*	0.62
SA vs. ENA	−2.341	0.0192*	0.46	−1.331	0.1833	0.26
PRA vs. LACA	1.399	0.1618	0.27	−0.246	0.8061	0.05
PRA vs. ENA	0.947	0.3437	0.19	2.204	0.0275*	0.43
LACA vs. ENA	0.769	0.4416	0.15	1.924	0.0544	0.38

Concerning pleasure, SA was likewise rated significantly lower than both LACA (*p* = 0.0023) and PRA (*p* = 0.0016), and PRA was rated significantly more pleasant than ENA (*p* = 0.0275). In contrast, pleasure ratings did not significantly differ between LACA and PRA (*p* = 0.8061), nor between LACA and ENA (*p* = 0.0544).

Effect sizes derived from the sample should be interpreted as approximate estimates of effect magnitude rather than precise or stable point estimates.

### Field notes content analysis

**Figure 1:**
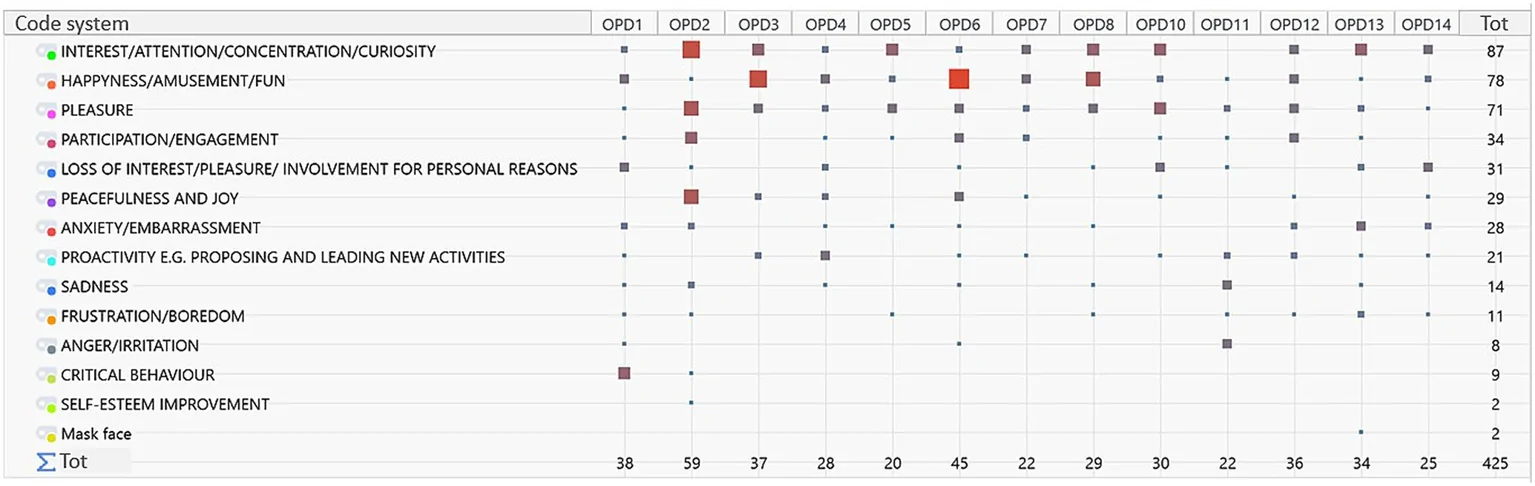
Code matrix frequencies by themes and cases. The size of the squares represents the number of times the codes were associated with fragments of the field notes. During the analysis, the association of two or more codes with the same text fragment was avoided.

The extraction of the frequencies of segments coded within the field notes of the external and internal observers (Figure 1) highlighted the prevalence of observed interest (87), happiness/amusement/fun (78) and pleasure (71), compared with other emotions, e.g., anxiety/embarrassment (28). In Figure 1, the size of the squares represents the number of times the codes were associated with fragments of the field notes. During the analysis, the association of two or more codes with the same text fragment was avoided.

The analysis of the content of the field notes taken by internal observers confirms that LACA and PRA were successful in increasing OPD’s interest/attention/concentration and pleasure:

*“G. is attentive: he moves together with the others following both slow and higher rhythmic music and keeps focusing on the balls”* (OPD5, male, 79); *“High pleasure in the activity with the sticks, during which he laughs heartily, and the one with “Volare”; briefer during the construction of the story”* (OPD8, male, 83); *“During the musical activity with the instruments, differently from the previous times, she chooses the instrument showing curiosity and seems to appreciate the activity. She plays a sound and tells the facilitator that she enjoyed using the chosen instrument”* (OPD13, female, 82).

Moreover, PRA including variations in speed and *tempo*, such as accelerations, decelerations, or expressive fluctuations, stimulated some participants’ proactivity more than SA and ENA, as illustrated by the following segment, where a rock tune stimulated OPD3 to invent a new rhythm:

*“During the instrumental song he independently proposes a rhythm with his legs. During the next Rock and roll song he continues to be active, very amused and involved to the point of proposing, again, new movements based on rock music. He also changes the rhythm, intensifying the strong accents”* (OPD3, male, 70).

Out of 13, 8 OPD (namely OPD1, OPD3, OPD4, OPD6, OPD7, OPD11, OPD12, and OPD13) became proactive in suggesting new movements, songs, and/or rhythms to the whole group. They took the initiative, acted intentionally, and enhanced group engagement and enjoyment, as well as their personal roles within the group, as illustrated by the following quotation:

*“She actively participates by responding to the facilitator in a prompt and interested manner, approaching her and proactively seeking contact by making comments”* (OPD12, female, 77).

On the contrary, ENA was associated with the greatest increase in OPD’s pleasure:

*“While listening to classical music by Vivaldi, A. continues to dance, stimulated and entertained by the music. She continues to dance on the chair and to chat. She is relaxed and listens to the co-created story, applauding at the end”* (OPD6, female, 81).

Nevertheless, listening activities involving purely calm and slow music, without associated body movements, often aroused sadness and melancholy in some participants because the music brought back memories of sad situations from the past:

*“I thought about too many things that are no longer here”* (OPD11, female, 84).

The following segment shows how experiencing pleasure can be associated with wellbeing:

“*While listening to relaxing classical music P. has a serene look and follows the agogics accompanying it with slow fluctuations of his hands and forearms, which he moves as if he is an orchestra conductor. When asked about what is coming to his mind, he says: “I was thinking of some kids who enjoyed listening to it… I’m feeling good here*” (OPD4, male, 84).

Other participants experienced pleasure by recognizing their ability to perform the activity well and by doing so with others:

*“He chooses again the instrument with the two cymbals: he is coordinated while playing it. The song “Dimmi quando tu verrai” is played and he follows the rhythm and stops when the facilitator does. He chooses the yellow harpsichord and performs the activity with great enjoyment, sings and at the end exclaimed: “We are very good!”* (OPD2, male, 77).

As mentioned previously, a range of emotions were observed, but only the most frequent are reported here, i.e., peacefulness/joy, anxiety/embarrassment, and anger/irritation, through the presentation of the most meaningful and representative experiences within the sample.

Feelings of peacefulness/joy were observed in OPD3, OPD4, OPD6, and OPD7, especially while they were listening to the iconic song “Volare” by Domenico Modugno. The way in which this music could transmit lightness and peace to participants and change their state of mind was impressive, as reported here:

“*He sings and claps his hands on the song “Volare” […] I feel very good! I felt light, I felt like I was flying”* (OPD4, male, 84) and *“Listening to the song “Volare” A. is relaxed and states “I feel lighter and younger”* (OPD6, female, 81).

Such a feeling of internal serenity made OPD7 more active and creative:

*“In the final greetings she sings joyfully, dances, claps her hands and thanks the facilitator: she is very involved in all the activities, with a great spirit of initiative and pleasure in being involved and in seeking contact with the facilitator”* (OPD7, female, 86).

The feeling of irritation (sometimes mixed with anger) prompted OPD1 to adopt a particularly critical and oppositional stance. The facilitator, who was the day-care center professional educator, was often able to defuse this behavior by involving her in activities, playing her favorite music, using her own communicative register, i.e., irony, and accompanying it with a smiling, reassuring, and welcoming attitude despite the participant’s sharp comments:

*“Then the facilitator distributes the image of children running in a park with balloons accompanied by a piece of music and asks them to listen and imagine what the children might be doing. At this point, the facilitator involves her in the story, telling everyone that they both come from the same town […]. At this point she smiles and, from this moment on, she participates more and smiles from time to time”* (OPD1, female, 83).

Over the course of the 12 sessions, OPD1 changed her attitude, becoming more cooperative and allowing herself to enjoy the experience of the SOUND intervention and music activities.

OPD11 was consistently an energetic, commanding, very directive woman who loved to be at the center of attention.

“*When the Twist begins and everyone is seated, [the facilitator] says: “Everyone stands up: move your legs, arms, head, go! We want to have fun. Body gestures suggest some anger and anxiety, when she makes gestures with her hands, grimaces or opens her eyes wide, sometimes she looks away, or has small tremors”* (OPD11, female, 84).

Feelings of anxiety/embarrassment were sporadically detected in some participants (OPD2, OPD4, OPD5, OPD6, OPD8, OPD12, and OPD14), especially when they were asked to answer a direct question or to perform a task with which they felt uncomfortable:

*“In the hula hoop activity, she smiles but it seems more out of her usual attitude of courtesy than out of enjoyment. She keeps her feet close together with her hands clasped in her lap and when she is asked to say the word that comes to her mind, she exclaims worriedly: Oh my God! She seems to show interest, but it is not clear whether this is due to concerns for her performance or genuine interest”* (OPD14, female, 93).

On the contrary, someone experienced a loss of attention when not being at the center of attention, when waiting for their turn, during the facilitator’s talks lasting more than 2 min, or when the activity became frustrating:

*“When talking about shopping (what would you put in the shopping cart, who does the shopping) he giggles, folds and handles the colored handkerchief, swings his feet while sitting, seems to want to move, and gets distracted in the spoken parts during which the facilitator’s turn is slow and a single participant can stay for a long time without being directly stimulated. In this moment his interest drops because he seems bored”* (OPD10, male, 81).

Differently, some comments concerned the pleasure of performing well together, as a group:

*“He calmly waited for the music, trying the instruments for about a minute, performing the suggested movements with interest, then exclaimed again “We’re good a lot”* (OPD2, male, 77).

Such a statement seems to acknowledge the group’s quality of performance and goes beyond the appreciation of individual performance, as if the feeling of being part of a group and the sense of community contributed to the enjoyment of the exercises and a greater perception of self-efficacy.

Regardless of the types of musical activities and the specific reactions of OPD, the researchers observed a progressive increase in interactions between the participants, with episodes of understanding, complicity, and empathy becoming increasingly frequent over the intervention, eventually resulting in hugs, laughter, knowing glances, smiles, and gestures of mutual encouragement:

*“While listening to the song “Volare” she sings and interacts with the gaze of OPD3 in front of her, takes the hands of the neighbors and claps her hands to the rhythm”* (OPD7, female, 86).

### Ideal types

Participants’ responses to each of the four macro areas of activity were analyzed to identify musical activities that most stimulated interest and pleasure and their attitudes toward them. We identified five ideal types of OPD exposed to musical stimuli: “Body-driven,” “Just singing,” “Reflective,” “Critics-anxious,” and “Enthusiastic.” In Table 3, the five ideal types are described, and segments from the field notes taken by internal observers are reported as evidence for the classification.

It is important to note that the “Enthusiastic” ideal type included all participants from the same group. The greater expression of interest and pleasure observed in this group may be related to several interacting factors, including the dynamics of collective participation, individual responsiveness to musical stimuli, facilitator characteristics, and contextual aspects of the intervention. Therefore, the relationship between this ideal type and group membership cannot be disentangled in the present sample, and differences associated with this type may partly reflect group-level characteristics rather than individual characteristics alone.

## Discussion and implications

In line with the literature (Dorris et al., 2021; Gómez-Gallego et al., 2021), the present findings suggest that different music-based activities may elicit different dimensions of engagement in older people with dementia. As shown by the Wilcoxon signed-rank test (Table 7), SA was significantly less interesting and pleasant than LACA and PRA, and less interesting than ENA, whereas PRA was significantly more pleasant than ENA.

However, a structural nuance should be considered when interpreting comparisons involving SA. Unlike PRA, ENA, and LACA, which generally formed extended core intervention modules, SA often functioned as framing elements (e.g., opening and closing greetings). Although the duration of singing tasks varied across sessions, the observation segments for SA may, in some instances, have been shorter than those of other categories, which could have subtly influenced how participant engagement was expressed and captured relative to longer activity blocks.

In the absence of direct neural measurements, a plausible, though speculative, explanation for why PRA were more enjoyable and engaging than other activities may relate to the neural networks involved, which tend to remain relatively preserved throughout the progression of neurodegenerative disorders. Rhythm perception and synchronization primarily involve sensorimotor systems and temporal processing networks that support motor coordination and embodied responses, which remain relatively accessible even in advanced stages of cognitive decline (von Schnehen et al., 2022). Early observational research also demonstrated that rhythm-based musical engagement remains feasible and meaningful even for individuals with severe dementia who show marked functional regression (Clair and Bernstein, 1995). Evidence from music therapy research also suggests that rhythmic and repetitive musical elements can regulate brain functioning, activate neural networks associated with memory and language, and support emotional regulation and social interaction (Lam et al., 2020; Bleibel et al., 2023). As such musical elements can be part of music activity programs led by musicians and/or healthcare professionals trained in music methodologies for people with dementia (not in music therapy), such as SOUND, similar effects may be observed and should be investigated further.

Furthermore, the results suggest that PRA might have provided an external temporal structure that facilitated interpersonal coordination, non-verbal communication, and emotional expression, thereby functioning as a form of cognitive scaffolding that supported attention, motor organization, and social participation in dementia care (Ghilain et al., 2020).

Notably, an increase in participants’ proactivity and creativity was observed when, while delivering PRA, the facilitators provided encouragement and constructive feedback, as this reinforced OPD’s willingness to become more explorative and take initiative in the group. Furthermore, the positive emotions experienced, together with the observation of peers’ proactive behavior, might have led to the imitation of such attitudes and increased creativity (Belschak and Hartog, 2026; Lieder and Iwama, 2021).

In contrast, ENA were less effective for some participants, probably because they required higher-order cognitive processes, including episodic memory, language processing, and executive functioning, which are more vulnerable to neurodegeneration (Falque et al., 2022; Kaltsa et al., 2024).

Concerning LACA, it is worth underlining that, since this macro-category groups distinct interventions, ranging from visual-motor coordination tasks (colored foulards and cloth with balls) to receptive auditory relaxation (calming music), high interest and pleasure ratings within LACA cannot be attributed unequivocally to a single specific task component. Future research utilizing component-dismantling designs is recommended to determine the relative efficacy of each constituent activity.

The analysis of the field notes showed consistency with the AARS analysis and also showed that negative emotions, such as anxiety and irritation, coexisted with positive ones, such as peacefulness, during the intervention. This may have been related to participants’ reduced coping resources and emotional regulation when facing difficulties experienced during the activities (Ross et al., 2022; Lou et al., 2024). This implies that the request to perform a new exercise may become a source of stress. In fact, the replication of exercises in a progressive overload manner helped participants build confidence and, for some, decrease anxiety over time, leaving room for amusement and proactivity (Santini et al., 2025).

The analysis also highlights the importance of the facilitators’ sensitivity and professionalism and the need to build a relationship of trust and affection with OPD, especially those experiencing anxiety and aggressiveness. The observation of the participants also confirmed that the use of selected cultural music (like “Volare” for Italian older adults) can be effective in stimulating positive emotions and thereby preparing OPD for an appropriate state of mind for improving cognitive function and mental health (Hyun-Sil and Jin-Suk, 2021), thanks to the persuasive power of music (Brancatisano and Thompson, 2020).

This result is in line with recent music-based interventions integrating culturally-anchored song choices and biographically tailored playlists to optimize therapeutic outcomes (García-Navarro et al., 2022; Hamiduzzaman et al., 2023). Because musical memory and emotional responsiveness often remain preserved even in advanced stages of neurodegenerative decline, leveraging songs rooted in a patient’s specific cultural, ethnic, and socio-historical identity activates autobiographical networks that standardized or generic “calming” playlists may fail to reach (García-Navarro et al., 2022). Research demonstrates that culturally tailored musical interventions significantly enhance engagement, foster affective stability, and alleviate neuropsychiatric symptoms such as agitation, anxiety, and sleep disturbances among community-dwelling and institutionalized individuals with dementia (García-Navarro et al., 2022; Hamiduzzaman et al., 2023; Petrovsky et al., 2020).

Furthermore, instrumental music activities, practiced in a small group setting, fostered social bonding, communication, shared experience, and enhanced social connection in OPD participating in the intervention, more so than group singing, as partly suggested by Bloska (2025).

### Translational implications for practice

The study underscored that a “one-size-fits-all” approach is not applicable to music-based interventions with OPD. Nevertheless, the identification of ideal types of OPD exposed to different musical stimuli suggests that mixed, short sessions including all planned activities should be tested to identify the most effective activities for triggering interest and pleasure, which could then be implemented in the intervention. Clustering OPD when testing the program may help identify activities suited to different types of OPD and design more effective music-based interventions. For example, LACA may be used with “Body-driven” participants, while ENA may be used with “Reflective” OPD.

The analysis of field notes from diaries also highlights that, generally, asking OPD to remember and verbalize something can be a source of embarrassment. This is not surprising, considering that dementia can affect language and memory from the very early stages of the disease. Therefore, musical activities should have more space than narrative ones in order to minimize the risk of provoking distress in OPD, which may lead to a lower effectiveness of the intervention.

In some individuals, certain types of exercises caused anxiety, which was always temporary and attributable to the reduced coping skills associated with their condition and to the exposure to the group context. This result may suggest that it would be beneficial to alternate between exercises of low difficulty and others that require greater effort to allow each participant to regain a state of comfort and confirm or recover a good level of self-esteem and self-efficacy. Anxiety may also be mitigated by the facilitator’s ability to accommodate the individual and the group, welcome participants’ inputs, refrain from judgment, and encourage participants to do the same. When this happened, in fact, we observed greater mutual support, participation, and spontaneity in the group.

In light of these results, it is important that the facilitator of similar interventions (i.e., those not requiring the involvement of a music therapist) is a healthcare professional who is familiar with the OPD’s life story, behavior, and health conditions and is part of a team including psychologists, social gerontologists, and musicians to properly design and deliver the intervention, categorize and group OPD based on the activities that most stimulate their interest and pleasure, interpret their reactions to the musical stimuli, and continually tailor/adapt the intervention to participants’ emerging needs, detected through the external and internal observers’ feedback and input. The five ideal types reported below (Table 8) are an attempt to categorize OPD into homogeneous clusters based on the music activities that most elicited their interest and pleasure, with the aim of informing the choice of music activities to maximize the benefit of the intervention.

**Table 8:** Suggestions for dementia care professionals by ideal type of OPD.

Ideal type of OPD	Suggestions for dementia care professionals
Body-driven	Primarily propose LACA and PRA: OPD need body movement as their main means of engagement. In contrast, avoid ENA, as story-telling and long verbal sessions may dampen interest and participation
Just singing	Primarily propose SA: play their preferred music so they relax and become cooperative and proactive. Avoid LACA and limit PRA: receptive listening may make them sad, while activities requiring coordination may be a source of stress
Reflective	Primarily propose ENA and avoid PRA and SA. They may be somewhat anxious and insecure and therefore prefer not to be at the center of attention: avoid asking them direct questions or asking them to perform an activity individually or to go first
Critics-anxious	Avoid LACA and propose ENA and PRA. Use the same communication style (e.g., irony), involve them as much as possible, play their preferred music, and adapt the plan to build rapport with them
Enthusiastic	PRA, SA, LACA, and ENA are all appropriate. You may even try to increase the complexity of the exercises. The group dimension is very important to them, and they are generally proactive and creative, so you can propose activities that involve interaction between individuals and the group in a responsive style

While speculative and untested in the absence of neural or formal measures of emotional contagion, the fact that the Enthusiastic type includes OPD from the same group might suggest, *post hoc*, a potential role for emotional contagion mechanisms (Hatfield et al., 1993), a hypothesis that warrants dedicated empirical testing in future studies.

### Study limitations and research suggestions

The study is not without limitations. First, the small sample size (13 participants) does not allow generalization of the results. Consequently, as also underlined in the Results, effect sizes derived from the sample should be interpreted as approximate estimates of effect magnitude rather than precise or stable point estimates. Second, since this pilot study did not include a control group, we cannot disentangle the specific contribution of the musical component from other contextual factors, such as increased social interaction, staff attention, novelty of the activity, or adaptation to the intervention setting, nor establish the extent to which the group and community dimensions might have influenced the participants’ pleasure and interest compared with the musical nature of the activity itself.

Furthermore, two additional methodological considerations warrant caution when interpreting our longitudinal findings. First, progressive improvements observed across sessions may partially reflect participant habituation to the research environment and to being observed, rather than a sustained intervention effect alone. Second, attrition-related selection bias cannot be ruled out; the two participants who dropped out prior to study completion may have systematically represented individuals who were more likely to have lower scores in earlier sessions, which could have artificially elevated the average scores in later sessions.

Third, even though the multiple methods of data collection helped identify the most interesting and pleasant activities, the study did not collect physiological parameters (e.g., heart rate and blood pressure) or biological data (e.g., cortisol, dopamine, oxytocin, or serotonin) that may be useful for detecting possible adverse effects, such as anxiety and stress.

Furthermore, the varying level of detail and accuracy in the field notes recorded by different observers might have influenced the data collection.

In addition, although live AARS observations were dynamically timed around key activity segments, retrospective video analysis was essential to complete scoring whenever live windows did not cover an activity’s full duration. To further streamline real-time data collection in future research, protocols could aim to implement full-length, continuous live tracking tailored directly to the precise onset and offset of each activity.

In addition to the above-mentioned limitations, other potential confounders could have influenced the outcomes: facilitator familiarity with participants; differences in facilitator background; the specific musical stimuli delivered; and baseline cognitive severity (a 3.2-point mean difference in MoCA scores between the two subgroups). Researchers adopted the following measures to reduce the impact of the first three confounders. Differences in facilitator familiarity with OPD were mitigated by pairing participants with familiar internal observers to ensure consistent engagement. Regarding differences in facilitator background, although one facilitator was an internal professional educator at the day-care center while the other was an external expert, both facilitators underwent standardized training in the original intervention protocol, followed identical procedural guidelines, and co-prepared all sessions. Regarding the specific musical stimuli delivered, activity structures, session objectives, and musical genres were maintained consistently across groups. Minor adaptations were limited to individual song or artist selections to accommodate participant preferences without altering the core intervention framework. Nevertheless, these mitigating measures reduced but did not eliminate the confounding potential of the above factors, which cannot be quantitatively disentangled with the present design.

These limitations and biases inform suggestions for future research, which should include RCTs, enroll larger samples of OPD, and include medial and biological measures to compare sociopsychological with biological and clinical indicators of pleasure and stress and provide a multidisciplinary understanding of the topic. Additionally, further studies should compare group psychosocial interventions with and without music, and individual versus group music-based approaches, to determine whether, how, and to what extent the social dimension, such as group structure and dynamics (e.g., the relationship with other participants and with the facilitator), may influence the effect of music on OPD’s interest and pleasure. Finally, further studies are needed to understand the how, why, and when a music activity that initially stimulates interest and pleasure becomes a source of stress, discomfort, anxiety, or frustration in OPD.

## Conclusion

The study shows a possible association between the implementation of LACA and PRA and the expression of interest and pleasure among OPD participating in the study, suggesting that rhythm- and listening-based activities could be prioritized to maximize participants’ engagement and enjoyment when designing music-based interventions for people with dementia.

Nevertheless, the study findings should be interpreted cautiously, as the small sample size, the absence of a control group, and the observational design do not allow us to disentangle the specific contribution of activity type from other contextual factors, including facilitator characteristics, participants’ cognitive profiles, and the social dynamics of the sessions. The study therefore, does not claim to demonstrate a causal link between the type of musical activity and the response of older people with dementia. Rather, it provides an empirical contribution to dementia care professionals who use, or wish to use, music in care or rehabilitative practice, enabling them to make more informed choices regarding the type of music activities to offer.

The participants’ ideal types are intended to provide a unified interpretative lens for capturing key features of individual responses and supporting the development of more effective music-based interventions for people with dementia. The results may guide the development of future non-pharmacological interventions based on music.

## Data Availability

The raw data supporting the conclusions of this article will be made available by the authors, without undue reservation.

## References

[ref1] Belschak F. D. Hartog D. N. D. (2026). When leaders act as role models of proactive behavior. J. Bus. Psychol. 41, 339–354. doi: 10.1007/s10869-025-10060-5

[ref9001] Berkovic D. Macrae A. Gulline H. Horsman P. Soh S. E. Skouteris H. (2024). The delivery of person-centered care for people living with dementia in residential aged care: a systematic review and meta-analysis. Gerontologist. 64:gnad052. doi: 10.1093/geront/gnad052, 37144737 PMC11020247

[ref2] Berridge K. C. Robinson T. E. (2016). Liking, wanting, and the incentive-sensitization theory of addiction. Am. Psychol. 71, 670–679. doi: 10.1037/amp0000059, 27977239 PMC5171207

[ref3] Bleibel M. El Cheikh A. Sadier N. S. Abou-Abbas L. (2023). The effect of music therapy on cognitive functions in patients with Alzheimer’s disease: a systematic review of randomized controlled trials. Alz Res Therapy 15:65. doi: 10.1186/s13195-023-01214-9, 36973733 PMC10041788

[ref7] Bloska J. (2025). Treatment differentiation between group music therapy and recreational choir singing for people with dementia and depression living in residential care homes: structured video analysis of the interventions in the MIDDEL trial. Front. Psych. 16:1730949. doi: 10.3389/fpsyt.2025.1730949, 41608470 PMC12835769

[ref8] Bowling D. L. (2023). Biological principles for music and mental health. Transl. Psychiatry 13:374. doi: 10.1038/s41398-023-02671-4 PMC10695969 38049408

[ref9] Bowling D. L. Ancochea P. G. Hove M. J. Fitch W. T. (2019). Pupillometry of groove: evidence for noradrenergic arousal in the link between music and movement. Front. Neurosci. 12:1039. doi: 10.3389/fnins.2018.01039, 30686994 PMC6335267

[ref10] Bowling D. Gahr J. Ancochea P. G. Hoeschele M. Canoine V. Fusani L. . (2022). Endogenous oxytocin, cortisol, and testosterone in response to group singing. Horm. Behav. 139:105105. doi: 10.1016/j.yhbeh.2021.105105, 34999566 PMC8915780

[ref11] Brancatisano O. Baird A. Thompson W. F. (2019). A ‘music, mind and movement’ program for people with dementia: initial evidence of improved cognition. Front. Psychol. 10:1435. doi: 10.3389/fpsyg.2019.01435, 31379638 PMC6646671

[ref12] Brancatisano O. Thompson W. F. (2020). “Seven capacities of music that underpin its therapeutic value in dementia care,” in Music and Dementia: From Cognition to Therapy, eds. Baird A. Garrido S. Tamplin J. (USA: Oxford University Press), 41–64.

[ref13] Clair A. A. Bernstein B. (1995). Rhythm playing characteristics in persons with severe dementia including those with probable Alzheimer’s type. J. Music. Ther. 32, 113–131. doi: 10.1093/jmt/32.2.113

[ref14] Dorris J. L. Neely S. Terhorst L. VonVille H. M. Rodakowski J. (2021). Effects of music participation for mild cognitive impairment and dementia: a systematic review and meta-analysis. J. Am. Geriatr. Soc. 69, 2659–2667. doi: 10.1111/jgs.17208, 34008208 PMC8440389

[ref15] Falque A. Jordanis M. Landré L. Loureiro de Sousa P. Mondino M. Furcieri E. . (2022). Neural basis of impaired narrative discourse comprehension in prodromal and mild dementia with lewy bodies. Front. Aging Neurosci. 14:939973. doi: 10.3389/fnagi.2022.939973, 36185488 PMC9520572

[ref16] Fancourt D. Finn S. (2019) What is the Evidence on the role of the arts in Improving Health and well-Being? A Scoping Review Copenhagen WHO Regional Office for Europe. Health Evidence Network (HEN) Synthesis Report 67. Available online at: https://iris.who.int/server/api/core/bitstreams/e1cc8536-773d-446f-9822-8ae376f41415/content (Accessed August 25, 2026). 32091683

[ref17] Forman J. Damschroder L. (2008). “Qualitative content analysis,” in Empirical Methods for Bioethics: A Primer, eds. Jacoby L. Siminoff L. A. (Amsterdam: Elsevier), 39–62.

[ref18] García-Navarro E. B. Buzón-Pérez A. Cabillas-Romero M. (2022). Effect of music therapy as a non-pharmacological measure applied to Alzheimer’s disease patients: a systematic review. Nurs. Rep. 12, 775–790. doi: 10.3390/nursrep12040076, 36278769 PMC9624344

[ref19] Ghilain M. Schiaratura L. Singh A. Lesaffre M. Samson S. (2020). “Is music special for people with dementia?” in Music and Dementia: From Cognition to Therapy, eds. Baird A. Garrido S. Tamplin J. (Oxford: Oxford University Press), 24–40.

[ref20] Golafshani N. (2003). Understanding reliability and validity in qualitative research. Qual. Rep. 8, 597–606. doi: 10.46743/2160-3715/2003.1870

[ref21] Gómez-Gallego M. Gómez-Gallego J. C. Gallego-Mellado M. García-García J. (2021). Comparative efficacy of active group music intervention versus group music listening in Alzheimer's disease. Int. J. Environ. Res. Public Health 18:8067. doi: 10.3390/ijerph18158067, 34360360 PMC8345612

[ref22] Gómez-Gallego M. Gómez-García J. (2017). Music therapy and Alzheimer's disease: cognitive, psychological, and behavioural effects. Neurologia 32, 300–308. doi: 10.1016/j.nrl.2015.12.003 26896913

[ref23] Hamiduzzaman M. Kuot A. Greenhill J. Strivens E. Parajuli D. R. Isaac V. (2023). Person-centred, culturally appropriate music intervention to improve psychological wellbeing of residents with advanced dementia living in Australian rural residential aged care homes. Brain Sci. 13:1103. doi: 10.3390/brainsci13071103, 37509033 PMC10377712

[ref24] Hatfield E. Cacioppo J. T. Rapson R. L. (1993). Emotional contagion. Curr. Dir. Psychol. Sci. 2, 96–100. doi: 10.1111/1467-8721.ep10770953

[ref25] Hsieh H. F. Shannon S. E. (2005). Three approaches to qualitative content analysis. Qual. Health Res. 15, 1277–1288. doi: 10.1177/1049732305276687, 16204405

[ref26] Hyun-Sil K. Jin-Suk K. (2021). Effect of a group music intervention on cognitive function and mental health outcomes among nursing home residents: a randomized controlled pilot study. Geriatr. Nurs. 42, 650–656. doi: 10.1016/j.gerinurse.2021.03.012 33823423

[ref27] Ito E. Nouchi R. Dinet J. Cheng C. H. Husebø B. S. (2022). The effect of music-based intervention on general cognitive and executive functions, and episodic memory in people with mild cognitive impairment and dementia: a systematic review and meta-analysis of recent randomized controlled trials. Healthcare 10:1462. doi: 10.3390/healthcare10081462, 36011119 PMC9408548

[ref28] Jakob E. Meininger J. Hillebrand M. Jakob E. Weise L. Wilz G. (2024). Study protocol: randomized controlled trial of an individualized music intervention for people with dementia in the home care setting. BMC Psychiatry 24:230. doi: 10.1186/s12888-024-05697-0, 38532365 PMC10967058

[ref29] Jiménez-Palomares M. Garrido-Ardila E. M. Chávez-Bravo E. Torres-Piles S. T. González-Sánchez B. Rodríguez-Mansilla M. J. . (2024). Benefits of music therapy in the cognitive impairments of Alzheimer's-type dementia: a systematic review. J. Clin. Med. 13:13. doi: 10.3390/jcm13072042, 38610807 PMC11012733

[ref30] Kaltsa M. Tsolaki A. Lazarou I. Mittas I. Papageorgiou M. Papadopoulou D. . (2024). Assessing the linguistic capacity across Alzheimer's disease and its preclinical stages: evidence from narrative macrostructure in elderly speakers of Greek. J. Alzheimer's Dis 100, S25–S43. doi: 10.3233/JAD-240496, 39121121

[ref31] Koelsch S. (2020). A coordinate-based meta-analysis of music-evoked emotions. NeuroImage 223:117350. doi: 10.1016/j.neuroimage.2020.117350, 32898679

[ref32] Kohlbacher F., (2006). The use of qualitative content analysis in case study research. Forum Qual. Soc. Res., 7. Available online at: http://www.qualitativeresearch.net/index.php/fqs/article/view/75/154 (Accessed August 25, 2026).

[ref33] Kutsche P. (1998). Field Ethnography: A Manual for Doing Cultural Anthropology. Upper Saddle River, NJ: Prentice Hall.

[ref34] Lam H. L. Li V. T. V. Laher I. Wong R. Y. (2020). Effects of music therapy on patients with dementia: a systematic review. Geriatrics 5:62. doi: 10.3390/geriatrics5040062, 32992767 PMC7709645

[ref35] Lawton M. P. Van Haitsma K. Perkinson M. A. Ruckdeschel K. (1999). Observed affect and quality of life in dementia: further affirmations and problems. Aging Ment. Health 5, 69–81.

[ref36] Leggieri M. Thaut M. H. Fornazzari L. Schweizer T. A. Barfett J. Munoz D. G. . (2019). Music intervention approaches for Alzheimer's disease: a review of the literature. Front. Neurosci. 13:132. doi: 10.3389/fnins.2019.00132, 30930728 PMC6424022

[ref37] Li X. Feng X. Sun X. Hou N. Han F. Liu Y. (2022). Global, regional, and national burden of Alzheimer's disease and other dementias, 1990–2019. Front. Aging Neurosci. 14:937486. doi: 10.3389/fnagi.2022.937486, 36299608 PMC9588915

[ref38] Lieder F. Iwama G. (2021). Toward a formal theory of proactivity. Cogn. Affect. Behav. Neurosci. 21, 490–508. doi: 10.3758/s13415-021-00884-y, 33721229 PMC8208939

[ref39] Lin T. H. Liao Y. C. Tam K. W. Chan L. Hsu T. H. (2023). Effects of music therapy on cognition, quality of life, and neuropsychiatric symptoms of patients with dementia: a systematic review and meta-analysis of randomized controlled trials. Psychiatry Res. 329:115498. doi: 10.1016/j.psychres.2023.115498, 37783097

[ref40] Lincoln Y. S. Guba E. G. Pillotta J. J. (1985). Naturalistic Inquiry. Thousand Oaks: SAGE.

[ref41] Liu M. N. Liou Y. J. Wang W. C. Su K. C. Yeh H. L. Lau C. . (2021). Group music intervention using percussion instruments to reduce anxiety among elderly male veterans with Alzheimer disease. Med. Sci. Monit. 27:e928714. doi: 10.12659/MSM.928714, 33611334 PMC7905960

[ref42] Lou Y. Vu T. Piechota A. Monin J. K. (2024). Emotion regulation in people living with dementia and their spouses: the role of neuropsychiatric symptoms. Aging Ment. Health 28, 1733–1740. doi: 10.1080/13607863.2024.2367038, 38940472 PMC11560677

[ref43] Luborsky M. R. Rubinstein R. L. (1995). Sampling in qualitative research: rationale, issues, and methods. Res. Aging 17, 89–113. doi: 10.1177/0164027595171005, 22058580 PMC3207270

[ref44] Matziorinis A. M. Koelsch S. (2022). The promise of music therapy for Alzheimer’s disease: a review. Ann. N. Y. Acad. Sci. 1516, 11–17. doi: 10.1111/nyas.14864, 35851957 PMC9796133

[ref45] Mayring P., (2000). Qualitative content analysis. Forum Qual. Soc. Res. 1. Available online at: http://www.qualitative-research.net/index.php/fqs/article/view/1089/2386 (Accessed August 25, 2026).

[ref46] McMahon K. Clark I. N. Stensæth K. Wosch T. Odell Miller H. Bukowska A. . (2023). A qualitative systematic review of the experiences of sharing music for people living with dementia and their family care partners: the thread of connection. Arts Health. 15, 229–256. doi: 10.1080/17533015.2022.2128381, 36224535

[ref47] Mulia G. J. (2025). Cognitive enhancement through music therapy: meta-analytic evidence across clinical population. Front. Public Health 13:1735470. doi: 10.3389/fpubh.2025.1735470, 41584142 PMC12823501

[ref48] Nasreddine Z. S. (2026) MoCA Cognition: Frequently Asked Questions/ Interpretation of the MoCA/What are the Severity Levels for the MoCA. Available online at: https://mocacognition.com/faq/ (accessed August 5, 2026)

[ref49] Nasreddine Z. S. Phillips N. A. Bédirian V. Charbonneau S. Whitehead V. Collin I. . (2005). The Montreal cognitive assessment, MoCA: a brief screening tool for mild cognitive impairment. J. Am. Geriatr. Soc. 53, 695–699. doi: 10.1111/j.1532-5415.2005.53221.x, 15817019

[ref50] Petrovsky D. V. Gooneratne N. S. Bradt J. Gitlin L. N. Hodgson N. A. (2020). Tailored music listening intervention to reduce sleep disturbances in older adults with dementia: research protocol. Res. Nurs. Health 43, 557–567. doi: 10.1002/nur.22081, 33136301 PMC7945958

[ref9003] Quattrini S. Merizzi A. Caciula I. Napradean L. João Azevedo M. Sandra C. . (2024). The design and implementation of a novel music-based curriculum for dementia care professionals: The experience of SOUND in Italy, Portugal and Romania. *BMC Med. Educ.* 24:668. doi: 10.1186/s12909-024-05651-4, 38886706 PMC11184888

[ref51] Quinci M. A. Belden A. Goutama V. Gong D. Hanser S. Donovan N. J. . (2022). Longitudinal changes in auditory and reward systems following receptive music-based intervention in older adults. Sci. Rep. 12:11517. doi: 10.1038/s41598-022-15687-5, 35798784 PMC9261172

[ref52] Ray K. D. Götell E. (2018). The use of music and music therapy in ameliorating depression symptoms and improving well-being in nursing home residents with dementia. Front. Med. 5:287. doi: 10.3389/fmed.2018.00287, 30356835 PMC6190855

[ref53] Reschke-Hernández A. E. Gfeller K. Oleson J. Tranel D. (2023). Music therapy increases social and emotional well-being in persons with dementia: a randomized clinical crossover trial comparing singing to verbal discussion. J. Music. Ther. 60, 314–342. doi: 10.1093/jmt/thad015, 37220880 PMC10560009

[ref54] Robinson T. E. Berridge K. C. (1993). The neural basis of drug craving: an incentive-sensitization theory of addiction. Brain Res. Rev. 18, 247–291. doi: 10.1016/0165-0173(93)90013-P, 8401595

[ref55] Ross S. D. Hofbauer L. M. Rodriguez F. S. (2022). Coping strategies for memory problems in everyday life of people with cognitive impairment and older adults: a systematic review. Int. J. Geriatr. Psychiatry 37:5. doi: 10.1002/gps.5701, 35362220

[ref56] Salimpoor V. N. Benovoy M. Larcher K. Dagher A. Zatorre R. J. (2011). Anatomically distinct dopamine release during anticipation and experience of peak emotion to music. Nat. Neurosci. 14, 257–262. doi: 10.1038/nn.2726, 21217764

[ref57] Salimpoor V. N. Van Den Bosch I. Kovacevic N. McIntosh A. R. Dagher A. Zatorre R. J. (2013). Interactions between the nucleus accumbens and auditory cortices predict music reward value. Science 340, 216–219. doi: 10.1126/science.1231059, 23580531

[ref9002] Santini S. Merizzi A. Caciula I. Azevedo M. J. Hera A. Napradean L. . (2024) A quasi-experimental mixed-method pilot study to check the efficacy of the “SOUND” active and passive music-based intervention on mental wellbeing and residual cognition of older people with dementia and dementia professionals’ burnout: a research protocol. Front. Psychol. 15:1327272. doi: 10.3389/fpsyg.2024.1327272, 38420177 PMC10901113

[ref9004] Santini S. Merizzi A. Azevedo M. J. Costa S. Caciula I. Di Rosa M. . (2025). Effects of a person-centered music-based intervention in the rehabilitation of older adults with mild to moderate dementia. J. Alzheimers. Dis. Rep. 9:25424823251367291. doi: 10.1177/25424823251367291 PMC12437183 40963978

[ref58] Shenton A. K. (2004). Strategies for ensuring trustworthiness in qualitative research projects. Educ. Inf. 22, 63–75. doi: 10.3233/EFI-2004-22201

[ref59] Skoda O. Conant M. Couture V. Bruneau M. A. Forget M. F. Nguyen Q. D. . (2026). Neuropsychiatric symptoms and caregiver burden in dementia: a longitudinal dyadic cohort study. Can. J. Neurol. Sci. 29, 1–27. doi: 10.1017/cjn.2026.10545, 41607071

[ref60] Stake R. E. (2005). Multiple Case Study Analysis. New York: Guilford Press.

[ref61] von Schnehen A. Hobeika L. Huvent-Grelle D. Samson S. (2022). Sensorimotor synchronization in healthy aging and neurocognitive disorders. Front. Psychol. 17:838511. doi: 10.3389/fpsyg.2022.838511 PMC8970308 35369160

[ref62] Wang Y. N. Wen X. N. Chen Y. Xu N. Zhang J. H. Hou X. . (2024). Effects of movement training based on rhythmic auditory stimulation in cognitive impairment: a meta-analysis of randomized controlled clinical trial. Front. Neurosci. 18:1360935. doi: 10.3389/fnins.2024.1360935, 38686327 PMC11057238

[ref63] Weber M. (1949). “Max Weber on the methodology of the social sciences,” in Methodologies of the social sciences, eds. Shils E. A. Finch H. A. (Glencoe, Illinois: Free Press).

[ref64] Wensing M. Eccles M. Grol R. (2005). “Observational evaluations of implementation strategies,” in Improving Patient Care: The Implementation of Change in Clinical Practice, eds. Grol R. Wensing M. Eccles M. (Edinburgh: Elsevier), 248–255.

[ref65] Yin R. K., (2009). Case Study Research: Design and Methods. 4th. Applied Social Research Methods Series. Sage, Thousand Oaks, CA.

[ref66] Zhang J. Yu Z. Zhang N. Zhao W. Wei B. He R. . (2023). Does music intervention relieve depression or anxiety in people living with dementia? A systematic review and meta-analysis. Aging Ment. Health 27, 1864–1875. doi: 10.1080/13607863.2023.2214091, 37243671

